# Supplementary material for: Paracoccidioides HSP90 Can Be Found in the Cell Surface and is a Target for Antibodies with Therapeutic Potential
Source: J Fungi (Basel). 2020 Sep 28;6(4):193. doi: 10.3390/jof6040193 (PMC7712200; doi:10.3390/jof6040193)
Supplement: Supplementary file 1 [file jof-06-00193-s001.zip › Supplemental figure legends.docx]

**Figure S1 – Anti-HSP90 polyclonal serum tested against fungal cytosolic or surface protein extracts, and on mammalian cell protein extracts. (A)** SDS-PAGE of cytosolic protein extracts. **(B)** WB shows the antiserum binds the target proteins in fungal cytosolic extracts. **X Axis: 1)** *E. coli* BL21a DE3 **2)** THP1 **3)** J774 **4)** Pb01 **5)** Pb18 **6)** SC3514 **7)** H99. **(C)** SDS-PAGE of surface protein extracts. **(D)** WB shows the antiserum binds the target proteins in fungal cell surface extracts. **X Axis: 1)** Pb01 **2)** Pb18 **3)** SC3514 4) H99. MW: Molecular Weight standard. The bands that possibly correspond to HSP90 are highlighted in red.

**Figure S2 – HSP90 immunolocalization in yeasts with polyclonal antisera.** Yeast cells were incubated with anti-HSP90 polyclonal sera from mice immunized with the recombinant protein, or with antisera from mice injected with 1% PBS as negative control. Detection was made with an Alexa Fluor 488-conjugated secondary Anti-IgG antibody. Fluorescence signals indicate presence of HSP90 on the surface and in the cytosol. **A)** *P. lutzii* **(B)** *P. brasiliensis* **C)** *C. neoformans* **D)** *Candida albicans*. Panels A-D show a single epifluorescence image, whereas panel E show a Z-projection of the deconvolved stack for each of the previous panels, to show the staining pattern more clearly. Scale bar: 10 µm.

**Figure S3 – HSP90 immunolocalization in *P. lutzii* and *P. brasiliensis* with hybridoma culture supernatants.** Yeast cells were incubated with supernatant from the 4D11 or 2C2 anti-HSP90 hybridoma, or with 1% PBS (NC) Negative Control. Detection was made with an Alexa Fluor 488-conjugated secondary Anti-IgG antibody. Fluorescence signals indicate presence of the proteins on the cell surface and in the cytosol. **A)** *P. lutzii* **(B)** *P. brasiliensis*. Scale bar: 10 µm.
